# Estimating and forecasting the burden and spread of Colombia’s SARS-CoV2 first wave

**DOI:** 10.1038/s41598-022-15514-x

**Published:** 2022-08-09

**Authors:** Jaime Cascante-Vega, Juan Manuel Cordovez, Mauricio Santos-Vega

**Affiliations:** 1 Universidad de los Andes, Grupo de Biología y Matemática Computacional (BIOMAC), Bogotá D.C., 111711 Colombia; 2 Present Address: Facultad de Medicina, Universidad de los Andes, Bogotá D.C., Colombia

**Keywords:** Computational models, Viral infection

## Abstract

Following the rapid dissemination of COVID-19 cases in Colombia in 2020, large-scale non-pharmaceutical interventions (NPIs) were implemented as national emergencies in most of the country’s municipalities, starting with a lockdown on March 20th, 2020. Recently, approaches that combine movement data (measured as the number of commuters between units), metapopulation models to describe disease dynamics subdividing the population into Susceptible-Exposed-Asymptomatic-Infected-Recovered-Diseased and statistical inference algorithms have been pointed as a practical approach to both nowcast and forecast the number of cases and deaths. We used an iterated filtering (IF) framework to estimate the model transmission parameters using the reported data across 281 municipalities from March to late October in locations with more than 50 reported deaths and cases in Colombia. Since the model is high dimensional (6 state variables in every municipality), inference on those parameters is highly non-trivial, so we used an Ensemble-Adjustment-Kalman-Filter (EAKF) to estimate time variable system states and parameters. Our results show the model’s ability to capture the characteristics of the outbreak in the country and provide estimates of the epidemiological parameters in time at the national level. Importantly, these estimates could become the base for planning future interventions as well as evaluating the impact of NPIs on the effective reproduction number ($\mathscr {R}_{eff}$) and the critical epidemiological parameters, such as the contact rate or the reporting rate. However, our forecast presents some inconsistency as it overestimates the deaths for some locations as Medellín. Nevertheless, our approach demonstrates that real-time, publicly available ensemble forecasts can provide short-term predictions of reported COVID-19 deaths in Colombia. Therefore, this model can be used as a forecasting tool to evaluate disease dynamics and aid policymakers in infectious outbreak management and control.

## Introduction

Coronavirus disease 2019 (COVID-19) pandemic emerged in December 2019 caused by the virus SARS-CoV2^1,2^. This pandemic started in Wuhan-China, but it quickly spread to several countries worldwide^1^. This rapid global spread of SARS-CoV2 has caused an urgent need for readily-available forecasts of the Spatio-temporal transmission patterns to inform risk assessment and planning instances. For example, in Colombia, the novel coronavirus (SARS-CoV2) was initially reported in Bogota on March 6, 2020. Then, the virus has spread rapidly to several municipalities in the country, and as of October 29, 2020, about 71 municipalities reported more than 50 accumulated deaths. On March 20, 2020, the government declared a nationwide lockdown to prevent the spread of the virus throughout the country. After the first lockdowns, several non-pharmaceutical interventions, including case isolating, contact tracing, quarantine of exposed persons, social distancing, travel restrictions, school, churches, and workplace closures, were in place in Colombia to reduce transmission of the virus^1,3^. Although some of these measures are still in place, the intensity of these restrictions has changed over time due to reopening attempts, generating changes in mobility and activity patterns. Thus, assessing the temporal variation of transmission in real-time for different country regions based on human mobility becomes essential for evaluating the possible effects of reopening the country’s economy. Nowcasting and forecasting the COVID19 dynamic can also illustrate early possible periods or scenarios with high transmission intensity and ultimately help the public health system assess, intervene, and formulate public health policies.

In recent years, the interest in generating real-time epidemic forecasts to help control and manage infectious diseases has grown, prompted by a succession of global and regional outbreaks of infectious diseases such as Zika and Ebola^4–6^. The current availability of epidemiological and digital data streams, enhanced by process-based models that account for mechanisms such as climate, demography, and mobility, among other factors, can provide a basis to evaluate the impact and effectiveness of intervention strategies in changing environments^7–9^. Different studies have proposed various mechanistic and statistical approaches to forecasting seasonal and epidemic diseases^10,11^. The limitation of statistical models is that these approaches focus on associations and correlations in the epidemiological time-series data without addressing the mechanisms behind disease transmission dynamics^10,12^. This element could be resolved by mechanistic models based on biological mechanisms that underlie population-level disease transmission. However, forecasting with this kind of model is challenging given the difficulty of accounting for different sources of uncertainty^7,11,13–15^.

Recently, mathematical models and forecasting algorithms have been used to forecast and understand diseases such as Ebola^16^, influenza^6,7^ and dengue^17^, and recently significant research has used models to explain and project COVID19 dynamics. These forecasting approaches combine data assimilation methods (a technique where observational data are combined with output from a model to produce an optimal estimate of the evolving state of the system) with dynamical transmission models. The methods let estimating parameters in real-time and epidemiological quantities; therefore, their outputs would allow rapid assessment and decision-making. Moreover, forecasting infectious diseases is a valuable tool that helps understanding disease transmission dynamics and planning future interventions. However, it can have biases due to assumptions in the short term of the current disease dynamics^8,18^. In this paper, we used an epidemiological model to account for the disease dynamics of the SARS-CoV2 first wave in Colombia and iterated filtering algorithms to fit the parameters of our model to the reported data across municipalities. We estimated the transmission parameters of the disease and modeled the dynamics in every municipality with more than 50 cumulative deaths. This study aimed to determine parameter estimates to understand SARS-CoV2 space-time dynamics, combine these estimates with the model dynamics to generate and evaluate weekly real-time now-casting, and forecast COVID19, community, spread and mortality in Colombia.

## Methods

### Data description

**Figure 1:**
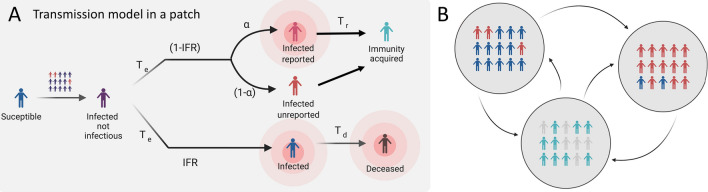
(**A**) Left: Cumulative observed cases of COVID19 by diagnosis date. (**A**) Right: Cumulative estimated cases by the nowcasting in the EAKF metapopulation model. (**B**) Left: Cumulative observed deaths of COVID19. (**B**) Right: Cumulative estimated deaths by the nowcasting in the EAKF metapopulation model.

We used daily reported cases (newly reported infections) by the Instituto Nacional de Salud (INS) in Colombia^3^. There each new infection is identified by a unique case ID and has an associated notification date by the surveillance system (SIVIGILA), the symptoms onset date (registered by the patient to the health care provider), and diagnosis date (documented by the laboratory after test confirmation). The epidemiological dataset also includes aspects such as recovery date, date of death, age, sex, municipality (county), department (state), type (imported from other countries versus associated, i.e., locally-acquired), location, if the patient is currently at home, hospital or ICU and the state/level of the disease (mild, medium or severe symptoms). We construct the daily community spread time series by confirmation date and mortality time series from this database (Fig. 1).

To incorporate the effect of human mobility between municipalities into our model, as depicted in Figure S2, we used movement data (measured as the number of reported commuters between units) from Facebook Mobility Data for Good. In addition, we used Facebook’s regular movement data, which aggregates the number of trips Facebook users make between every pair of municipalities over time^19^.

### Model description

We use a meta-population model, to model the transmission dynamics at each location following a SEAIIRD model. For simulating the community spread, we formulated the model as a discrete Markov process across days, and it assumes that susceptible individuals get infected at the rate $\lambda _i$ or force of infection (FOI). Following the mass law action, we consider the **FOI** is proportional to the number of contacts between susceptible individuals ($S_i$) and infectious reported individuals $I_i$ at rate $\beta _t$ and assume non-reported individuals to have relative transmissibility of $\sigma _tA_i$ they infect at rate $\sigma _t \beta _t$. The model subdivides infectious stages into three classes: **i)** Exposed (E): Infected but not infectious individuals, **i)** infected non-reported individuals (A): Infectious non-reported individuals (accounting mostly for asymptomatic transmission), and **i)** *Infected reported individuals.* We assume both infected reported ($I_r$) and non-reported (*A*) individuals infected for an average time of $T_r$ days before acquiring immunity.

**Figure 2:**
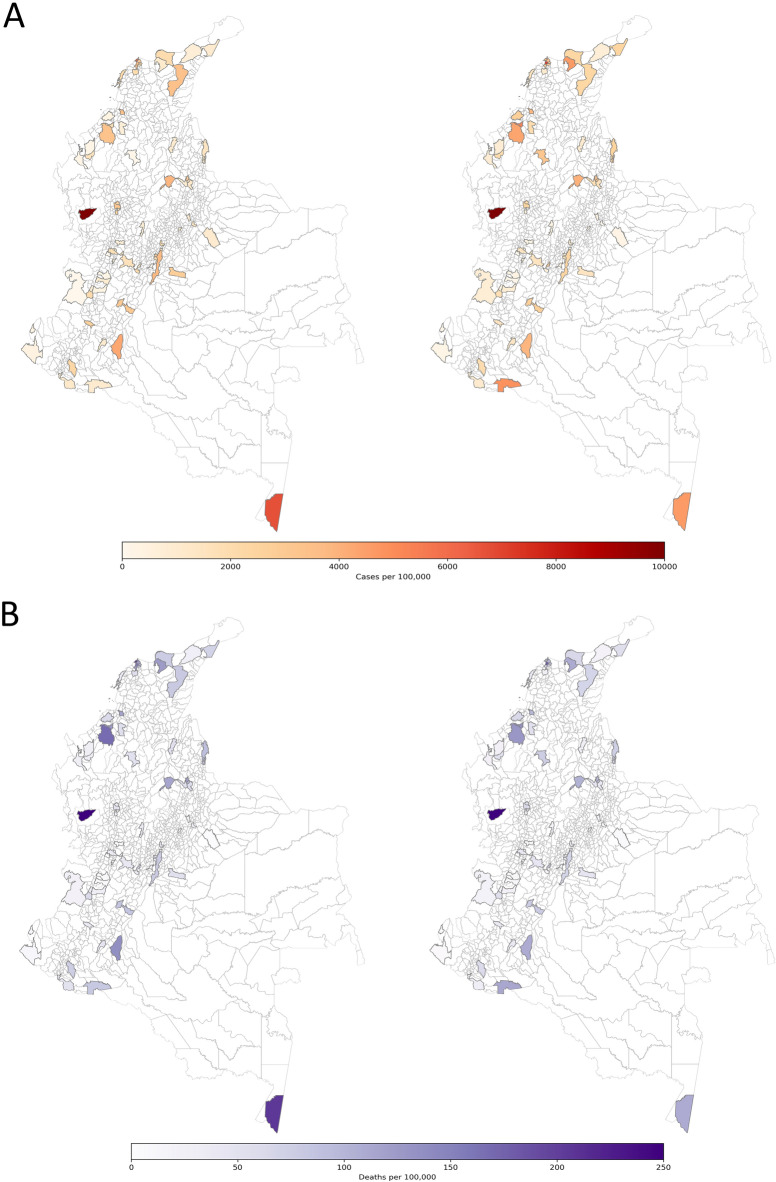
Meta-population SEAIIRD model. (**A**) Schematic representation of the spatially explicit epidemiological model in a patch of the population, where population is subdivided in Susceptible ($S_i$), Exposed ($E_i$), Unreported infections mostly accounting for asymptomatic or mild infections ($A_i$), Infected ($I_i$), Infected individuals that eventually are gonna die ($L_i$) and Recovered ($R_i$). This captures the local transmission dynamics in every municipality, importantly yellow compartments represent individuals who do not move within municipalities. (**B**) Schematic of meta-population model, connections between municipalities.

Our transmission model assumes multiple locations are connected by human mobility, then sub-populations of susceptible, exposed, unreported infected, reported infected, and recovered individuals move from municipality *i* to *j* at time *t*, and it is represented by $M_{ij}(t)$. We provide a **parameter** $\beta$ such as the municipal contact rate for transmission due to documented infected individuals. We assume that the transmission rate due to undocumented individuals is reduced by a factor $\sigma _i$. This relative transmissibility is based on the assumption that unreported individuals are mostly asymptomatic and have mild infections and therefore do not get tested^20^. In addition, $\alpha$ is the report fraction, or the proportion of total detected infections $I_i(t)$ individuals. $T_e$ is the incubation time in days (time from infection to symptom onset for symptomatic individuals), $T_r$ is the infectious period (time from symptom onset for symptomatic individuals or since exposure for asymptomatic individuals to recovery) also in days, and $T_d$ is the death period (time since exposure to death). Figure 2 shows the model diagram for the population dynamics of COVID-19.

*We use the 2020 Colombia’s national statistics demographic projections* for the total population of the $i-th$ municipality $N_i$^21^. The transmission model equations are shown below:

1 $$\begin{aligned}&\lambda _i(t) = \beta (t) \frac{I_i+ \sigma A_i }{N_i} \quad \text {Force of Infection} \end{aligned}$$

2 $$\begin{aligned}&\frac{dS_i}{dt}= -\lambda _i(t) S_i + \theta \sum _j \frac{M_{ij}(t) S_j }{N_j-I_j-L_i} - \theta \sum _j \frac{M_{ji}(t) S_i}{N_i-I_i-L_i} \nonumber \\&\frac{dE_i}{dt}= \lambda _i(t) S_i - \frac{E_i}{T_e}+ \theta \sum _j \frac{M_{ij}(t)E_j}{N_j-I_j-L_i} - \theta \sum _j \frac{M_{ji}(t) E_i}{N_i-I_i-L_i}\nonumber \\&\frac{dA_i}{dt}= (1-\zeta )(1-\alpha ) \frac{E_i}{T_e} - \frac{A_i}{T_r} + \theta \sum _j \frac{M_{ij}(t)A_j}{N_j-I_j-L_i} - \theta \sum _j \frac{M_{ji}(t) A_i}{N_i-I_i-L_i} \nonumber \\&\frac{dI_i}{dt}= (1-\zeta )\alpha \frac{E_i}{T_e} - \frac{I_i}{T_r} \nonumber \\&\frac{dL_i}{dt}= \zeta \frac{E_i}{T_e} - \frac{L_i}{T_d} \nonumber \\&\frac{dR_i}{dt}= \frac{A_i}{T_r}+\frac{I_i}{T_r} + \theta \sum _j \frac{M_{ij}(t) R_j}{N_j-I_j-L_i} - \theta \sum _j \frac{M_{ji}(t) R_i }{N_i-I_i-L_i} \end{aligned}$$

#### Mobility data and parametrization

Our model uses the information of the number of commuters to parametrize the equation below, where $X_i$ corresponds to the number of individuals in the epidemiological state *X* in (susceptible *S*, exposed *E*, asymptomatic *A* or recovered *R*) and municipality *i*. We assumed both infected reported $I_r$ individuals and infected who eventually are going to disease *L* do not travel between municipalities/patches and therefore are discounted in the denominator. This calculation divides the total commuters from patch *j* to path *i* by the fraction of individuals in each epidemiological state that we assume can commute. Partitioning the commuters from the Facebook Data for Good only requires accounting for commuters’ report rate parametrized by $\theta$.

3 $$\begin{aligned} M_{X_i} = \theta \frac{\sum _i M_{ji}X_i }{N_i-I_i-L_i} \end{aligned}$$

### Parameter estimation and forecasting

#### Parameter inference

We use the model to estimate non-observed epidemiological dynamics by fitting the model to the observed number of cases by confirmation date and deaths reported from March 06 to October 11, 2020, to estimate the model epidemiological parameters. We only estimated parameters for municipalities that reported more than 50 cumulative deaths by the first week of October. We use an Ensemble Adjustment Kalman Filter (EAKF), which applies to high dimensional models to assimilate daily data^7,13,22^.

Furthermore, we use an Iterative Filtering approach to infer model parameters and state variables; this iterated filtering (IF)-EAKF framework has been used to infer parameters in large-scale models as network metapopulation models for other pathogens^7,13,23,24^. We start by uniformly sampling from the prior ranges defined in Table S1. To address the limitation of the surveillance system report, we choose the prior fraction of reported cases $\alpha$ to cover almost all of its domain in [0,1]. A similar range was used for the relative asymptomatic transmission or unreported individuals, which have been shown to be primarily asymptomatic $\sigma$^13,18,25^. Importantly, in this case, we assume that the viral load in this sub-population cannot be greater than the viral load of reported individuals $I_i$ as has been assumed and estimated^1,25^. Finally, we also estimated the ascertainment or infection detection rates of Medellín using the same model structure without the metapopulation commuting model and found that it has substantial differences compared with the estimated for the country and attribute these differences to the overestimation.

We assume equal initial conditions for all municipalities regardless of the number of cases reported in the first days of SARS-CoV2 detection. We assume that each municipality starts with one infected individual and three exposed individuals $I_i(0)=1$, $E_i(0)=3$ and the seeding strategy follows the next protocol: The reporting delay in every municipality it is described by a Gamma distribution with with mean delay $P_{d_i}$ in days (see supplementary information Section 1). We then assume the seed of each municipality is $T_0 = t_i-P_{d_i}$ where $t_i$ is the date of the first reported case in municipality *i*. This seeding strategy follows the rationale that the first confirmed infection was seeded $P_{d_i}$ days before its confirmation.

#### Reporting delay

Our transmission model does not explicitly account for the time lag between the infection and their notification by PCR or Antigen test. However, we consider a delay between the symptoms onset report and the laboratory test result. For this, we mapped the simulated documented infections to the confirmed cases using a separate observation delay model to account for this notification delay. To estimate this lag period, $t_d$, we examined the observed distribution of the time interval to the event (in days) from the onset of the symptoms to confirmation and adjusted a Gamma distribution to it (Figure S1). In practice, in the transmission model simulation, for each new documented infection that goes from $E_i$ to $I_i$, a random number for $t_d$ with a Gamma distribution is generated. In other words, this case is “reported” as a confirmed infection $t_d$ days after the transition from $E_i$ to $I_{i,t}$. Therefore, the number of cases reported in one day accumulates as the model is integrated over time. Our model inference approach was applied to three different periods of the first pandemic wave of COVID19; first to the period before the strong lockdown (March 3rd to March 20th, 2020), then during the lockdown period (March 21th to May 1st, 2020), and finally the period of relaxation and progressively reopening (May 2nd to October 11th, 2020).

#### Seeding index cases

We initialize each spatial unit with the same number of infected and exposed individuals ($I_r$ and *E* in the model’s equations); we consider time heterogeneity in the seeds. The seeding strategy is as follows: each municipality has a computed Gamma distribution for the reporting delay, the mean delay $T_{d_i}$ in days, subscript indicating the municipality. We then assume the seed of each municipality is $T_0 = t_i-T_{d_i}$ where $t_i$ is the date of the first reported case in municipality i. This seeding strategy follows the rationale that every first confirmed infection is seeded $T_{d_i}$ days before its confirmation.

### Real time nowcasting and forecasting

We used the IF-EAKF to estimate time-varying parameters and variables and then used last week’s estimate to forecast future dynamics. Kalman Filters assume a Gaussian distribution for both the prior and likelihood. Therefore, the distribution of the system state can be fully parameterized by the first two moments (the ensemble mean and covariance)^7^. Based on this assumption, the posterior mean and covariance are calculated through the convolution of two Gaussian distributions. However, by generating the prior using a nonlinear model (e.g., the SEIIR model here), the model-filter system can estimate the nonlinear system dynamics despite the linear assumption of the Kalman filter algorithm.

For the EAKF, the ensembles are updated deterministically, then the ensemble mean and covariance match their theoretical values exactly. Then, the higher moments of the prior distribution are preserved in the posterior. The EAKF also adjusts the unobserved state variables and parameters based on their covariance with the observed state variables. EAKF is a suitable technique in problems like this because its implementation is independent of the dynamical model. This method allows both to simulate and make short-term predictions assuming particular scenarios which map to parameter space^22,23^. Here we used multiple observations from different locations to optimize the model by iterating over all observations sequentially and adjusting the entire state vector.

### Metrics

To assess model performance, we used different metrics to evaluate how the nowcasts and forecasts generated perform every week; then, we evaluated the performance of our nowcasts on a weekly horizon. For out-sample validation we project both number of cases and number of deaths assuming dynamics (parameter estimates) remained the same as the previous 10 days. This forecast reasoning has also seen in^26^ (See section S5 in Supplementary Material for further information). Next, we evaluated the forecast performance using different scores^27–29^. We investigate the probabilistic assessment of our forecast. We compute the sharpness, Bias and accuracy as measured by the Ranked Probability Score (RPS), Dawid-Sebastiani score (DSS) and Log-Score (LS). The scores, and it’s mathematical description can be seen in Table 1^5,29^. Finally, we split up the data into two subsets, the first one was used for testing, and the other subset was used for training the model’s, for evaluating the model performance in an out-of-sample way.

The *sharpness* is the ability of the model to generate predictions within a narrow range of possible outcomes. It is a data-independent measure, so it is purely a feature of the forecasts themselves, as shown in Table 1. To evaluate sharpness at time *t*, we used the normalized median absolute deviation about the median (MADN) of the prediction at time *t*^5^; this metric not only considers point errors as the mean square error or absolute error but has information on the posterior error median, that one would expect to be close to zero. Here, the model forecast performances were averaged across the weekly estimates and reported each month. We also assessed the **bias** to study if the model systematically over or under-predict. The forecast bias at time *t* is depicted in Table1^5^. An unbiased model would have $B_t\approx 0$ whereas an biased model would have $B_t>1$ if the model overestimate at time *t* and $B_t<-1$ if the model *under-predict* at time *t*. We say the model systematically over-predicted if $B_t>1$ averaging across the time series. Similarly, the model *under-predicts* if $B_t<-1$ in average. Finally, we evaluate a ranked probability score (RPS), which reduces to the mean absolute error if the forecast is deterministic^5^ and the coverage (CP) probabilities at confidence intervals of $95\%$ and $50\%$. This score considers the number of observations falling inside the specified model area^27^. We also computed the continuous Ranked Probability Score (RPS) which rather than providing a distance from a scalar prediction it measures the performance for a probabilistic prediction of a scalar observation. It is a quadratic measure of the difference between the prediction cumulative distribution function (CDF) and the empirical CDF of the observation.

**Table 1:** Summary and description of the metrics used for evaluating the quality of both nowcast and forecast and their performance. In these y is a variable with CDF $P_{t}$, and *X* and $X'$ are independent realizations of a random variable with cumulative distribution $P_{t}$.

Score	Measure	Equation	References
Median absolute deviation normalized (MADN)	Sharpness	$\frac{1}{0.675}\text {median}(|\mathbf {y_t}-\text {median}(\mathbf {y_t})|)$	^5^
Bias	Bias	$1-(P_t(x_t)+P_t(x_t-1))$	^5^
*Ranked probability score (RPS)*	Probabilistic Fit	$\sum _{k=0}^\infty (P_t(k)-\mathbb {I}(k\ge x_t))^2$	^5,28^
$\alpha$ *Ranked probability score (RPS-*$\alpha )$	Probabilistic Fit	$\sum _{k=0}^\infty (P_t(x_t)^{1-\alpha } \le P_t(x_t) \le P_t(x_t)^\alpha ))$	^5,28^
*Dawid-Sebastiani score (DSS)*	Probabilistic Fit	$\left( \frac{x_t-\mu _{P_t}}{\sigma _{P_t}}\right) ^2+2\log \sigma _{P_t}$	^5^
*Absolute error of the median (AE)*	Fit	$|\text {median}(P_t(X))-x_t|$	^5,27^
*Log Score (LS)*	Probabilistic fit	$\log (P_t(x_t))$	^27^

## Results

Parameter estimates for these three periods are reported in Table 2. Our estimates for the infectious period and latency period are $\sim 2.66$ days, consistent with the period estimated in the literature^13^. In addition, the transmission rate $\beta$ and the report rate $\alpha$ are consistent with values assumed by^1^ or estimated values by^13,30,31^.

**Table 2:** Estimated parameters in three different moments of the epidemic. Before country-level restrictions, during NPIs, and after relaxing NPIs. We assume the infectious period $T_r$, the incubation period $T_i$, and the death period $T_d$ of individuals are constant in time.

Parameter	Description	Units	Before lock-down	During lock-down	Lock-down relaxation
			Mean (95% CIs) 03-March–20-March	Mean (95% CIs) March 21th–1st-May	Mean (95% CIs) 2nd-May–11-October
$\beta$	Contact rate	Days$^{-1}$	1.066 (1.062, 1.081)	1.014 (0.994, 1.038)	0.993 (0.959, 1.012)
$\sigma$	Relative asymptomatic transmissibility.	-	0.465 (0.465, 0.465)	0.462 (0.462, 0.463)	0.463 (0.462, 0.465)
$\alpha$	Report fraction	-	0.339 (0.334, 0.351)	0.260 (0.244, 0.270)	0.303 (0.169, 0.414)
$\theta$	Movement report	-	1.361 (1.361, 1.362)	1.361 (1.360, 1.362)	1.361 (1.360, 1.362)

**Figure 3:**
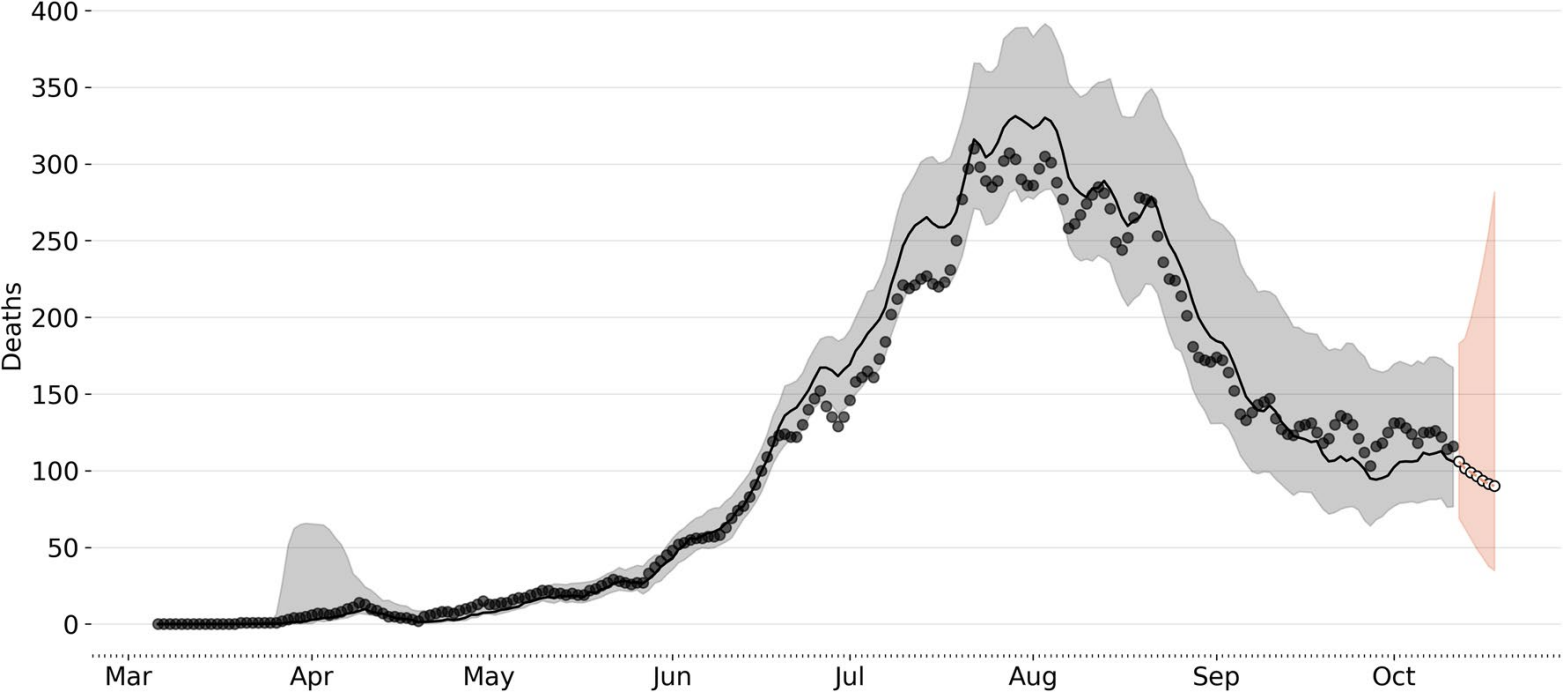
National Forecast (Aggregated municipal level forecast). This aggregation is the sum of all the deaths predicted for each municipality. Black line represents the median of the now-casting, the gray dark points are the daily deaths and the light gray area represents the $95\%$ confidence interval. The orange white-dotted line represents the forecast assuming the parameters as the mean of the last week. Again the light orange area represents $95\%$ confidence intervals.

Comparisons between model simulations and data are shown in Fig. 3 at the national level. This figure shows simulations of reported cases using the best-fitting model parameter estimates and their confidence intervals. These results from the stochastic simulations show that our model can capture the temporal dynamics of the epidemic. In addition, the best-fitting model captures the space-time pattern of COVID19 infections in different municipalities in Colombia, as shown in Fig. 3 and for the time pattern see Fig. 1.

**Figure 4:**
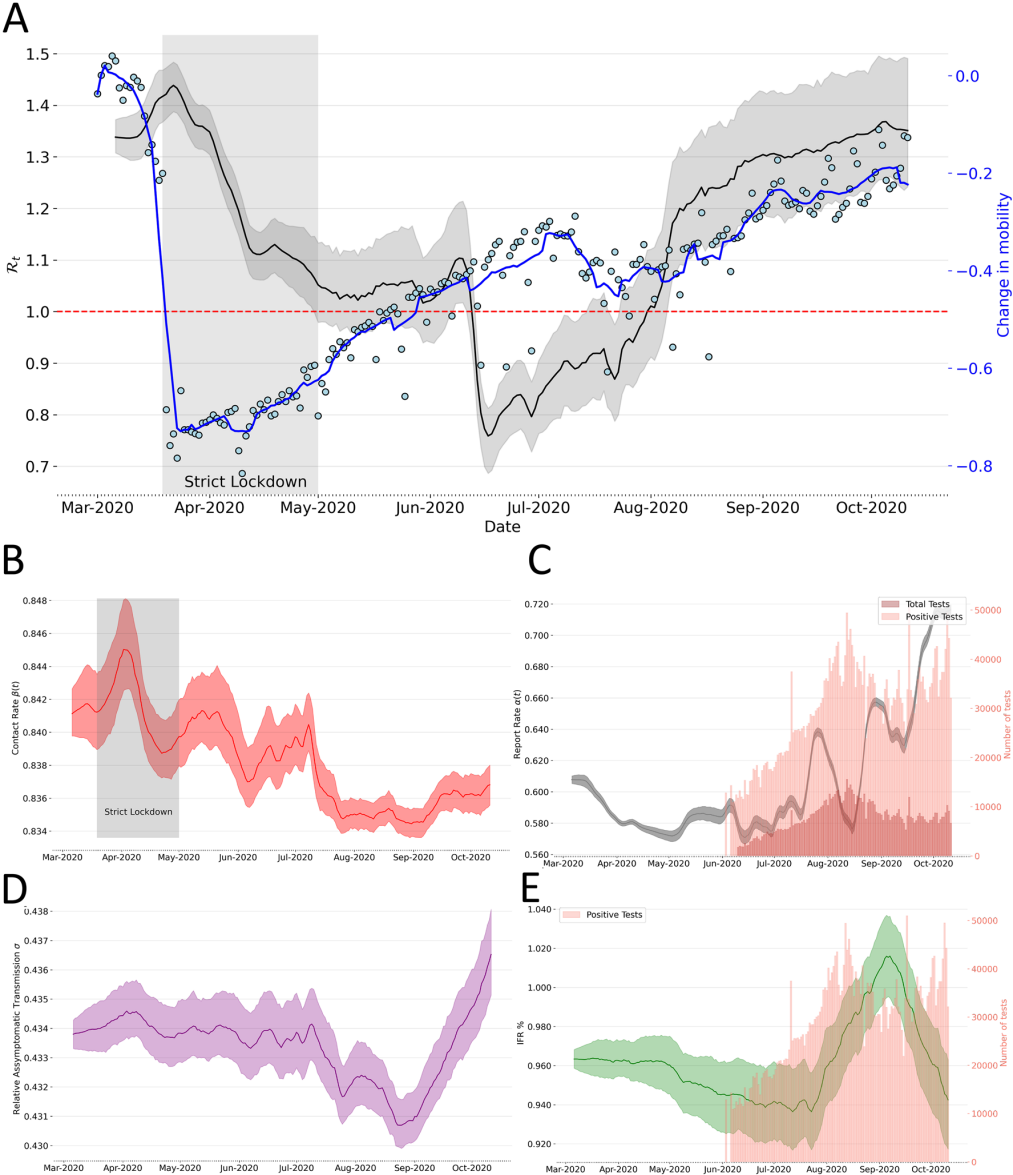
For all figures the lighter areas represent the 95% confidence interval and line represents the median estimate. (**A**) National effective reproduction number computed as the mean of every municipality $R_{eff}= \frac{1}{K}\sum _{i=1}^KR_{eff}^K$; lighter area represent the the $95\%$ confidence interval. (**B**) Time variable contact rate $\beta (t)$ lighter area represents the $95\%$ confidence interval. (**C**) National time variable report rate $\alpha$. (**D**) Relative Asymptomatic transmissibility $\sigma$. (**E**) Infection Fatality Risk (IFR %) $\zeta$.

Figure 4 presents our median estimate of the effective reproduction number ($R_{eff}$). This quantity is equivalent to the basic reproduction number, $R_0$, at the beginning of the epidemic *was around 2.24 [95 % credible interval (CI):* $2.21-2.32$], which coincides with the reported $\mathscr {R}_0$ for Colombia for COVID19. Indicating that this number has consistently been above 1 for COVID-19 in the country, suggesting a high capacity for sustained transmission (Table 2 and Fig. 4D). Significantly, reductions in $R_{eff}$ are associated with the lock-down measures during April, with sustained increases in this number after the reopening. Figure 4 also shows the value of the parameters on which $R_t$ depends ($\beta _t$, $\sigma \beta _t$ and $\alpha$). Noteworthy, time variation in the contact rates ($\beta =\beta _t$) closely matches the trajectory of deaths and the cases in the country. There is a decreasing trend in the number of detected infections and important variations in the fraction of asymptomatic cases, causing the most infection events. We can compute the effective reproduction number $R_{eff}$ as the case reproduction number $R_t$ times the fraction of susceptible individuals in the population $S_t/N$ where $N=\sum _i N_i$ for every municipality; this can be seen in Figs. S3 and S4.

**Figure 5:**
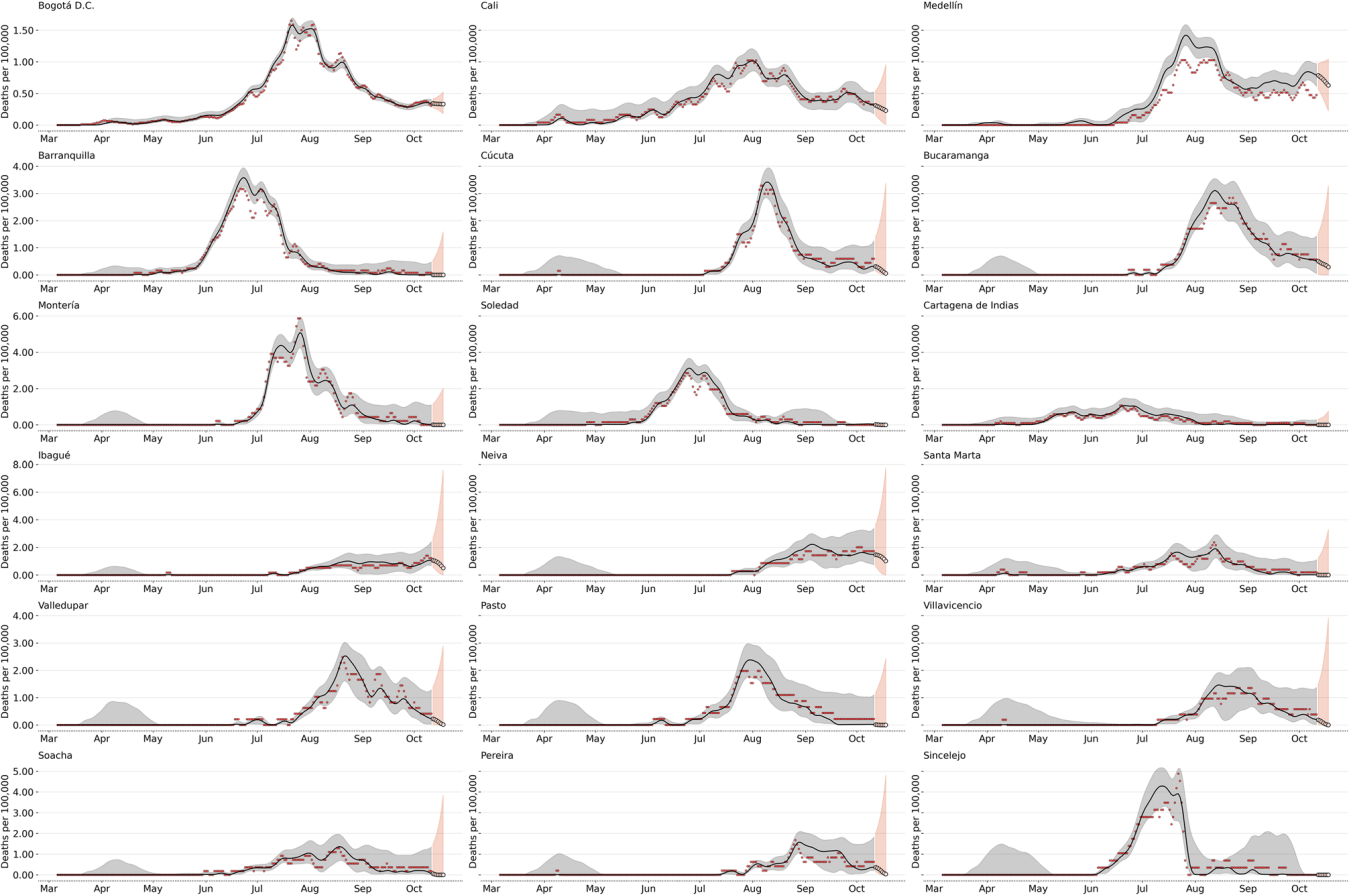
7 day death-forecast municipalities with more reported deaths by early October. Black line represents the median of the now-casting, the gray dark points are the daily deaths and the light gray area represents the $90\%$ confidence interval. The orange white-dotted line represents the forecast assuming the parameters as the mean of the last week. Again the light orange area represents $90\%$.

Figure 5 shows forecasting for different representative regions of Colombia (the remaining units are reported in the supplementary information). Our models can capture the temporal variation in the data at local scales, where most of the observed cases and deaths fall within the model’s confidence interval. The orange boundary shows weekly forecasting for the diseased; while our posterior estimates generally present a good fit to the observed mortality, we have some errors predicting it. For example, in Fig. 5 we can see an overestimation of the time series for the city of Medellín (depicted upper right). This pattern, in general, is consistent with the ability of the model to recreate the first wave in the country but highlights the considerable heterogeneity in reporting rates, for example, across space that is not considered the model, and other epidemiological differences as *fraction of infections that result in fatalities or infection fatality rate (IFR).*

Table 3 shows model scores aggregated over the top-10 locations. While Bias, DSS, and LS remain roughly constant, the MADN and RPS model scores increase over time. This is because both score metrics are based on the number of deaths. In fact increases in time in this metric are a consequence of changes in the number of deaths between May and October and not to a decrease in model sharpness or quality of the prediction. As cases increases o does mortality and the uncertainty associated with the prediction, resulting in higher MADN.

## Discussion

The proposed model-inference captures the spatiotemporal dynamics of COVID19 in Colombia, allowing us to generate a short-term forecast of the spread of the virus and the following number of deaths despite high heterogeneous transmission across the country. Forecasting the daily cases and deaths becomes important for prioritization and resource allocation by public health authorities. Also, our inference framework allows time variable parameter estimation, which is a valuable feature to characterize the evolution of the country’s local transmission dynamics and ultimately generate disease trajectories in the long term. We demonstrate that the standard epidemic SEAIR-type model under the assumption that homogeneous mixing of individuals is limited to account for the transmission dynamics observed for COVID19. The results of this study underscore the importance of short-term forecasts (one-week horizon) since the future of ongoing epidemics is sensitive to parameter values that change over time. Therefore, using this framework would be only meaningful within a narrow time window, even smaller than what we are used to in weather forecasts^32,33^. The median estimates for the latency and infectious period, $T_e$ is $\sim 3.4679$, and $T_r$ is $\sim 3.4324$ days; we fixed those parameters during the fitting process assuming generation interval remain fixed in time. The median estimate for the dead period is $\sim 11.822$ days. The ascertainment rate $\alpha$ in the country is estimated to be around $30\%$ of total infections reported. This estimate reveals a high rate of undocumented infections: $45\%$, as shown by different estimates around the globe^13^. Moreover, the estimated time variable parameters (view Fig. 4) generally agree with the estimated parameters in the literature^13^.

Over July, the model consistently predicted the number of cases in each municipality, although there is much higher variance in these weekly forecasts than in national forecasts. Furthermore, we demonstrated that using a transmission model with a meta-population structure incorporating population fluxes and accounting for the effect of commuting will significantly add information about the spatiotemporal dynamics. Traditionally this effect is usually contained in the contact rate $\beta$ at a population level. Also, we consider that our modeling framework considered a more realistic approach where spatial heterogeneity in factors such as time-varying disease onset times and the time-dependence of the contact rates are accounted^34^. In addition, we demonstrated the potential of sequential data assimilation for COVID-19 dynamics at a regional level and in combination with stochastic epidemiological models. Using an Iterated Filtering with an Ensemble Adjustment Kalman Filter (IF-EAKF), we successfully determined the contact parameter from simulated data and obtained reliable estimates from empirical data^22^. Notably, a characterization of the heterogeneity in the transmission parameter ($\beta$) is the most critical free parameter in our stochastic SEAIIRD model since other parameters (mean exposed and infectious duration of incubation period) can be extracted from the literature, given that are intrinsic parameters of the disease^13,35^.

Interestingly, since the transmission rate is estimated in time, and this parameter is directly related to the basic reproduction number $\mathscr {R}_0$^34^, our approach becomes a valuable method to infer the effective reproduction number ($\mathscr {R}_{eff}$). Our results show a decay in the $\mathscr {R}_{eff}$ from March to June; this coincides with the early lockdown period followed by a plateau which is explained by an increase **in both the time varying transmission rate** $\beta _t$ **as shown in Figure 3B and the mobility Figure shown below.** The decay in $\mathscr {R}_{eff}$ after May is a consequence of both the slowdown in the mobility change as well as a plateau in the number of new spatial units with reported cases between May 1st and June 5th. We have studied the difference in the effective reproductive number to respect the parameters (the sensitivity index of $\mathscr {R}_t$ of alpha, for example, accounts for the report rate). Rather than comparing absolute changes, we have normalized the sensitivity indices to compare the 1% changes of parameters to see how it influences $\mathscr {R}_{eff}$. The analytical expression of the sensitivity indexes is shown in SI Section S9. We found that the parameters that affect the most $\mathscr {R}_{eff}$ are the transmission rate $\beta$, recovery period $T_r$ with a sensitivity index of 1, and the report rate $\alpha$ with a sensitivity index of 0.443. This result also highlights the importance of estimating these quantities in time, reflecting the underlying community structure that affects transmission with $\beta$ and the surveillance system’s effectiveness in capturing unseen infections or $\alpha$.

Our results are the first estimates for the country and the only estimation of the under-reported or asymptomatic infections. These findings provide a baseline in Colombia to assess the fraction of undocumented infections and their relative infectiousness. In addition, these results describe the transmission dynamics in Colombia, a Latin American country, one of the regions with the highest attack rates . Our approach allows us to evaluate the impact of changes in interventions, viral surveillance, and testing on the reported fraction. As shown in Fig. 4C, we found an average report rate of $38\%$, which results in around $60\%$ of undocumented infections. This trend also follows the country’s testing and positivity rate trends. However, it is essential to highlight that this fraction shows their dependency on the time of the peak, the surveillance effort, and the spatial heterogeneity.

A recent evaluation of forecasts in the United States has shown the importance of including probabilistic and point estimate metrics to assess forecast accuracy (distance to observed data) and quality (coverage of forecast distribution)^26^. After evaluating the forecasting performance with different score measures, our epidemiological model and the inference method can accurately predict the number of cases in the country one week in advance, as reported results by month in Table 1. Interestingly some big urban centers like Medellin depicted in Fig. 5 upper right overestimate the fitted posterior incident deaths. To understand these differences between Medellín and the rest of Colombia, in Figure S4, we show Medellin’s sub-reporting compared to the national one. We see that differences are principally accounted for by discrepancies in the case-ascertainment rates or reporting rates that we assumed were constant across the country.

Our work, however, has some limitations that are important to highlight. First, pre-symptomatic individuals might have a higher viral load than symptomatic ones hence infecting more; yet, we do not explicitly model pre-symptomatic transmission. Although different models assume asymptomatic individuals infect less than symptomatic ones, transmission model parameter estimation also suggests this. **Finally, municipalities** could only be forecasted if they had a minimum of 50 deaths. In addition, it must be noted and considered during the interpretation of these results that, first, the model is optimized using observations of both communities spread by confirmation date and mortality data through October 11, 2020, a range in which communities across different cities were under different NPIs as city and other spatial level lockdowns. Second, even when we use mobility data to disaggregate the effect of mobility between municipalities, the considerable space-time variability in epidemic seeding, epidemic timing, and testing practices makes fitting any model challenging. Yet, it is also important to highlight that the effects of NPIs will not be seen after 10–14 days after the interventions took place. Considering infections that the surveillance systems model does not capture, many asymptomatic, mild, and other more severe infections did not seek testing. Therefore, this is a crucial part of the sources of uncertainty in the model since we did not incorporate testing records explicitly. While the variance in a forecast prediction is a common feature of most COVID19 forecasting models, it appears to be quite large in our forecast.

**Table 3:** Scores for evaluating probabilistic forecasts. The table depicts monthly values of (MADN), (Bias), (RPS), (DSS), and (LS) from May to October 2020 to evaluate the predictive performance of the model.

Month	MADN	Bias	RPS	DSS	LS
May	0.109	0.76	52.19	11.88	− 6.86
June	0.213	0.77	30.31	12.02	− 6.92
July	0.344	0.76	14.60	11.96	− 6.90
Aug	0.494	0.72	17.57	11.88	− 6.86
Sep	0.695	0.66	55.39	11.85	− 6.85
Oct	0.98	0.63	104.29	12.09	− 6.96

It is expected that uncertainty in the forecasts would provide more insights into the COVID19 pandemic trajectory, modeling, and forecasting malpractice. The considerable uncertainty in the prediction shows that the trends can change rapidly, principally by the difficulties of predicting people’s behaviors in response to the different epidemiological scenarios and public health/government policies and how this is real-time changing the contact rates interacting with the current epidemiological state^32^. More data or specific studies would be needed to reduce the uncertainty of the estimates. In addition, data protection issues and real-time gathering of data impose challenges. The forecast results are shown in Fig. 3, and Fig. 5 shows an increasing declining trend in the incident cases. Nonetheless, the observed trend was stable for that period and increased in the first days of December. This highlights an important limitation of dynamic transmission models, that as they have a threshold $\mathscr {R}_{eff}$ uncertainty around $\mathscr {R}_{eff}=1$ does not allow models to reproduce stable trends in deaths. Moreover, our model is implemented as a stochastic Markov chain process and therefore uncertainty is inherent to the stochastic nature of the system.

The proposed model has four crucial assumptions on the SARS-CoV2 spread: We directly assume the delay from the infection to the report date fitting a Gamma distribution as shown in Figure S1 in Supplementary Material. This assumption relates to the challenge of reconstructing the time series of new infections, as observations occur long after transmission. The model and the parameter inference setting let us estimate time-variable contact rates for both reported individuals and asymptomatic/mild infections, which directly account for the mobility restrictions imposed to reduce the transmission. The model also assumes a time-variable asymptomatic/mild infections fraction, accounting for the possible high number of asymptomatic infections. About the limitations of our model, as we describe in the “Methods” section, our model does not track differences in the associated patch for each individual and the patch they reside in at time T. This mismatch is a product of both the model we use and the available aggregated and anonymized mobility data from Facebook Data for Good to compute the number of commuters between municipalities/patches at daily time steps. In addition, our modeling framework heavily relies on the quality of the surveillance data to make estimates of the fraction of the reported cases or the testing capacity, among others. So, it is crucial to mention that places with weak surveillance systems could lead to forecasts that are not as good and show high uncertainty.

Our work underscores the importance of mechanistic models for explaining spatio-temporal dynamics of COVID19 and therefore estimate time-varying parameters that allow recreating the transmission and further understanding of relevant epidemiological features of transmission across scales. While using a dynamical system to describe the disease dynamics and have a mechanistic understanding of the system, we recognize that purely statistical methods that use past trends in the data to project the time series might be more effective to forecast the future^27^. Many statistical models were designed to be either more flexible or parsimoniously parameterized, meaning that they may more easily capture dynamics typical to infectious disease time series, such as dynamics dependent on the previous consecutive time point and seasonality. In the case of this emergent pandemic, where limited data is available, mechanistic models may be able to take advantage of assumptions about the underlying transmission process, enabling rudimentary forecasts even with minimal data^27^. It is important to consider that making predictions just one week into the future will need to account for the non-linear nature of infectious diseases, which makes possible future scenarios incredibly uncertain. For example, minor initial differences in infection parameters can lead to significant differences in outcomes with time- and it certainly seems plausible that this makes it challenging to estimate one’s level of certainty^36^. Previous experiences with influenza forecasting have demonstrated that it is often possible to quantify uncertainty over the remainder of an ongoing flu season. However, this success was primarily based on observing the behavior of seasonable epidemics over several decades^37^. To reliably forecast the progression of pandemics, where relevant historical data are almost nonexistent, we must have a detailed quantitative understanding of how different, diverse factors affect disease transmissibility. Importantly communicating forecasts in the COVID-19 pandemic exhibit considerable challenges and trade-offs in communicating uncertainty concerning public trust. For example, occasionally downplaying uncertainties may strengthen public trust in the short term, but confident predictions that later turn out wrong may reduce public trust in science^36^. Then it is crucial to consider these aspects to make forecasting operational (e.g., communicate about these forecasts publicly), consider broad ranges of possible outcomes as plausible, and communicate this high level of uncertainty to non-experts. In summary, while statistical methods based purely on observed trends in the data are helpful to forecast short-term dynamics, their accuracy in early stages (few data points available) and their lack of mechanistic understanding of the system makes their mechanistic model’s counterpart desirable. That said, we should thoroughly validate forecasts in the early stages of their development since they rely heavily on assumptions of the disease dynamics system. In conclusion, our approach becomes a valuable tool for the country to understand the dynamics and estimate effects with comparatively little data at the level of regions. Importantly, future interventions should combine the benefits of the models’ estimating parameters with analysis tools to deploy NPIs in a specific area better.

## Supplementary Information

Supplementary Information.

## Data Availability

The datasets generated and/or analysed during the current study are available in the Github repository, https://github.com/biomac-lab/sarscov2_colombia_estimates.
