# Supplementary material for: Estimating and forecasting the burden and spread of Colombia’s SARS-CoV2 first wave
Source: Sci Rep. 2022 Aug 9;12:13568. doi: 10.1038/s41598-022-15514-x (PMC9427755; doi:10.1038/s41598-022-15514-x)
Supplement: Supplementary file 1 — Supplementary Information. [file 41598_2022_15514_MOESM1_ESM.pdf]

Supplementary Material for

Estimating and forecasting the burden and spread of Colombia's SARS-CoV2 first wave.

Jaime Cascante-Vega, Juan Manuel Cordovez, Mauricio Santos-Vega

Universidad de los Andes, Grupo de Biología y Matemática Computacional (BIOMAC), Bogotá D.C., 111711, Colombia

Supplementary Material

S.1 Basic Reproductive Number

The model equations are given by:

$$\lambda_i(t) = \beta(t)\frac{I_i + \sigma A_i}{N_i} \quad \text{Force of Infection} \tag{1}$$

$$\begin{aligned}
\frac{dS_i}{dt} =& -\lambda_i(t)S_i + \theta\sum_j \frac{M_{ij}(t)S_j}{N_j - I_j - L_i} - \theta\sum_j \frac{M_{ji}(t)S_i}{N_i - I_i - L_i}\\
\frac{dE_i}{dt} =& \lambda_i(t)S_i - \frac{E_i}{T_e} + \theta\sum_j \frac{M_{ij}(t)E_j}{N_j - I_j - L_i} - \theta\sum_j \frac{M_{ji}(t)E_i}{N_i - I_i - L_i}\\
\frac{dA_i}{dt} =& (1-\zeta)(1-\alpha)\frac{E_i}{T_e} - \frac{A_i}{T_r} + \theta\sum_j \frac{M_{ij}(t)A_j}{N_j - I_j - L_i} - \theta\sum_j \frac{M_{ji}(t)A_i}{N_i - I_i - L_i}\\
\frac{dI_i}{dt} =& (1-\zeta)\alpha\frac{E_i}{T_e} - \frac{I_i}{T_r}\\
\frac{dL_i}{dt} =& \zeta\frac{E_i}{T_e} - \frac{L_i}{T_d}\\
\frac{dR_i}{dt} =& \frac{A_i}{T_r} + \frac{I_i}{T_r} + \theta\sum_j \frac{M_{ij}(t)R_j}{N_j - I_j - L_i} - \theta\sum_j \frac{M_{ji}(t)R_i}{N_i - I_i - L_i}
\end{aligned} \tag{2}$$

For computing the Basic Reproductive Number we use the Next Generation Method (NGM) which account for changes in states where individuals have the virus E, A, I, L we compute the basic reproductive number as follows, we define the transition rates $\mathcal{F}$ and removal rates V.

$$\mathcal{F} = \begin{bmatrix} \beta S \frac{I+\sigma A}{N} \\ (1-\zeta)(1-\alpha)\frac{E}{T_e} \\ (1-\zeta)\alpha\frac{E}{T_e} \\ \zeta\frac{E}{T_e} \end{bmatrix} \quad \mathcal{V} = \begin{bmatrix} E/T_e \\ A/T_r \\ I/T_r \\ L/T_d \end{bmatrix}$$

We then compute the transition and removal matrix F and V respectively as follows. And evaluate at the disease free equilibrium $S = N$.

$$F = \begin{bmatrix} 0 & \beta\sigma & \beta & 0 \\ (1-\zeta)(1-\alpha)/T_e & 0 & 0 & 0 \\ (1-\zeta)\alpha/T_e & 0 & 0 & 0 \\ \zeta/T_e & 0 & 0 & 0 \end{bmatrix} \quad V = \begin{bmatrix} 1/T_e & 0 & 0 & 0 \\ 0 & 1/T_r & 0 & 0 \\ 0 & 0 & 1/T_r & 0 \\ 0 & 0 & 0 & 1/T_d \end{bmatrix}$$

$$FV^{-1} = \begin{bmatrix} 0 & \beta\sigma T_r & \beta T_r & 0 \\ (1-\zeta)(1-\alpha) & 0 & 0 & 0 \\ (1-\zeta)\alpha & 0 & 0 & 0 \\ \zeta & 0 & 0 & 0 \end{bmatrix}$$

Then the basic reproductive number computed as the maximum eigen-value of FV^{-1}.

$$\mathcal{R} = \mathcal{R}_u + \mathcal{R}_r$$

Where we define $\mathcal{R}_u$ as the expected secondary infection due to under-reported individuals (recall that this we also believe this contribution is mostly due to asymptomatic infections which by definition are the individuals less captures by the surveillance system) and $\mathcal{R}_r$ as the expected secondary infection due to reported individuals. This is consistent with the term involved in the proposed Force of Infection (FOI) in Equation 1, which only assume that unreported and reported individuals infect susceptible ones. Note that for the reproductive number do to under-reported individuals $\mathcal{R}_u$ the fraction $(1-\zeta)(1-\alpha)$ is the expected fraction of population entering the A compartment, here $zeta$ is the infect fatality risk and $alpha$ the fraction of reported individuals, where $\sigma\beta$ is their transmission or contact rate and T_r is the average time expected in the A compartment. Similar for the reproductive number do to reported individuals $\mathcal{R}_r$ we have $(1-\zeta)\alpha$ is the expected number of individuals entering the I compartment and β is their transmission rate and T_r is the expected time before acquiring immunity.

$$\mathcal{R}_u = \sigma\beta T_r(1-\zeta)(1-\alpha), \quad \mathcal{R}_r = \beta T_r(1-\zeta)\alpha$$

As we assume the contact rate is time variable $\beta = \beta(t)$ we then compute the effective reproductive number in municipality i as, where $S_i(t)$ is the posterior sample of the estimated value of susceptible individuals at time t and N_i is the population in the municipalite i:

$$R^i_{eff}(t) = \mathcal{R}(t) \cdot \frac{S_i(t)}{N_i}$$

We then compute the national effective reproductive number as, for each one of the 71 municipalities that have reported more than 71 deaths by the date.

$$R_{eff}(t) = \frac{1}{71} \sum_{i=1}^{71} R_{eff}^{i}(t)$$

S.2 Report Delay Distributions

We fit a Gamma distribution to the difference in days between symptom onset date and diagnosis date for addressing the report delay natural of the surveillance system using the SCIPY package available in Python [1]. The figure below shows some examples of the fitted Gamma distribution for the capitals of states/departments in Colombia. Note that using the fitted gamma distribution worth to model as probably by surveillance system or diagnosis laboratory reports an unusual number of cases is reported usually at 15 days. Therefore we recall the importance of used a fitted distribution rather than the empirical measured one. The importance of modeling this report delay have also been highlighted in current best practices for estimating the time varying effective reproductive number [2, 3].

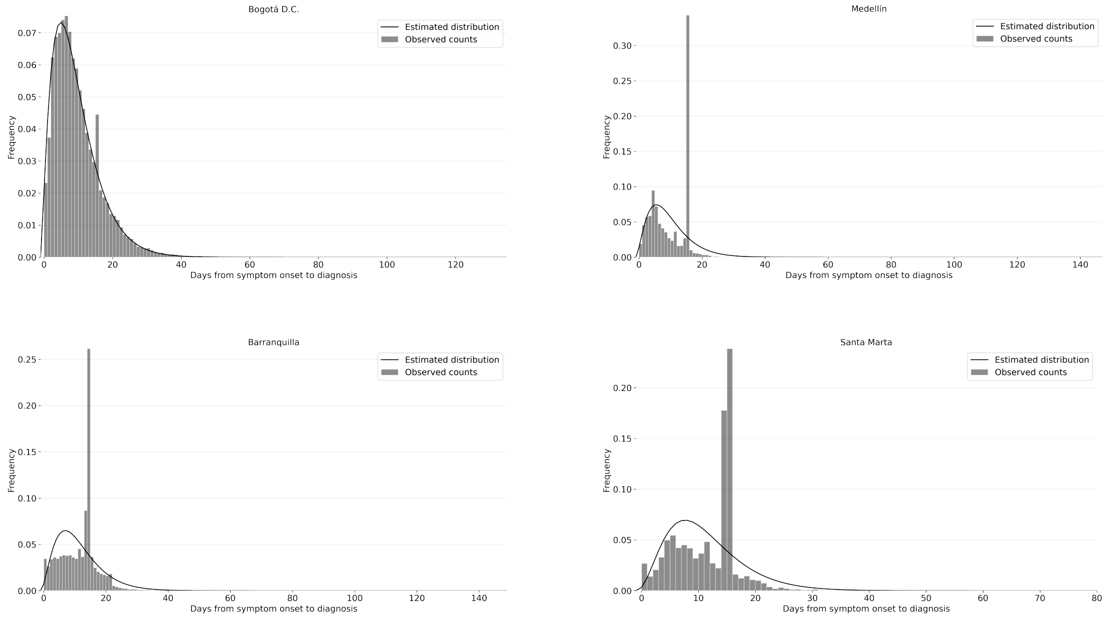

Figure S1: Fitted delay distributions for some municipalities. As seen is more frequent that the data is reported with a specific delay between symptom onset and diagnosis at 15 or 16 days (about 2 weeks) which we believe is due to return of diagnosis by the laboratories. Therefore we recall the importance of used a fitted distribution rather than the empirical measured one.

S.3 Facebook Mobility Data

Commuters evolution from Facebook mobility data mobility in four different time periods.

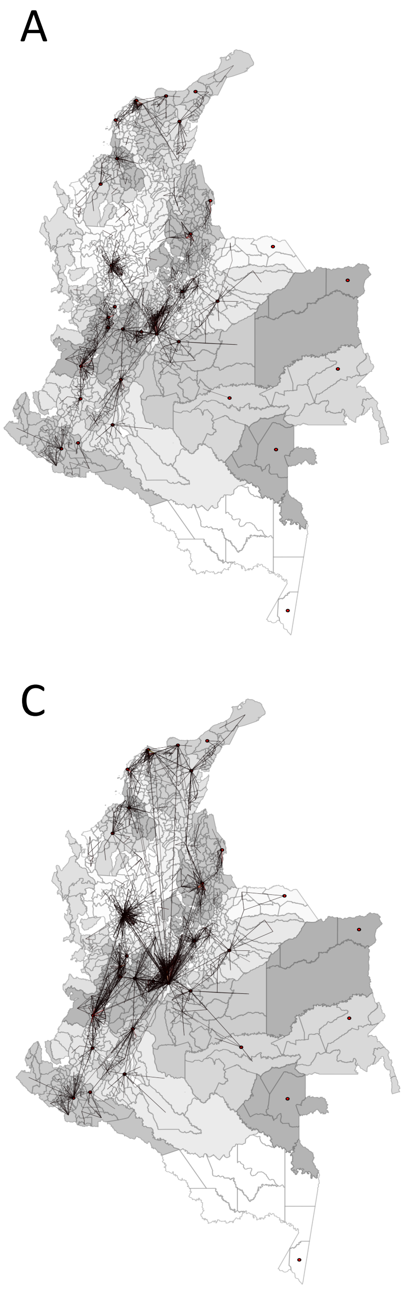

Figure S2: Facebook Mobility Data between municipalities, black lines represent commuters between a pair of municipalities and red dots are capitals of states/department of Colombia. States/Department are color-keyed as gray-scale in the map. Each map represent a specific time periods selected according to the national non-pharmaceutical interventions (NPIs) imposed in the country. **A** Average number of commuters in the strict national lockdown from 01-April-2020 (first available data) to 31-May-2020 first national reopening. **B** Average number of commuters from 01-June-2020 to 31-July-2020, two months after national re-opening. **C** Average number of commuters from 01-August-2020 to 31-October-2020, two month period four months after national re-opening. **D** Average number of commuters after 31-October-2020.

S.4 Parameter Inference

For estimating the key local epidemiological parameters and therefore characterizing the disease spread of COVID19 we use flat uninformed priors as shown in Table S1. For the infect fatality risk parameter (IFR) ζ we search over all the IFR range reported for different countries and across ages [4, 5].

Parameter	Prior Distribution	Reference
β	$\sim \mathcal{U}(0.75, 1.2)$	-
α	$\sim \mathcal{U}(0.01, 1)$	-
T_e	$\sim \mathcal{U}(2, 5)$	[6]
T_r	$\sim \mathcal{U}(2, 5)$	[7, 6]
θ	$\sim \mathcal{U}(0.5, 1.25)$	[7]
T_d	$\sim \mathcal{U}(7, 15)$	
ζ	$\sim \mathcal{U}(0.1\%, 2\%)$	-

Table S1: Uninformative prior distribution on the estimated parameters for the epidemiological meta-population model

S.5 Operational Forecasting

For forecasting $t > T$ where T is the last fitted day for all municipalities. We do not attempt to model or impose assumptions about future change in behavior that might impact the contact rate β or assume for example that testing is increasing resulting in increased report fraction α. We simply use the estimated values of the time varying parameters prior to the forecast horizon (we simple assume parameters will remain constant in the future). However for modeling temporal variation of behavior and based on the belief that behavioral changes occur smoothly in time we average over the last 10 days instead of using the last day estimates. We attempt to use more than one week as estimates might be impacted by report model of the surveillance system as has been showed in [2]. This approach have been similarly followed in [8]. We then assume for time variable parameters $\theta(t)$ that their value in the forecast horizon t_f follows Equation 3. Moreover for embedding this into the EAKF framework we treat each ensemble separately, therefore conserving parameter estimates within each ensemble member.

$$\theta(T+i) = \frac{1}{10}\sum_{i=1}^{10}\theta(T-j) \quad \forall i \in [T, T+t_f] \tag{3}$$

S.6 Time estimates of the effective reproductive number

Median estimates of the effective reproductive number R_{eff} for the top 5 municipalities with more reported cumulative death by October.

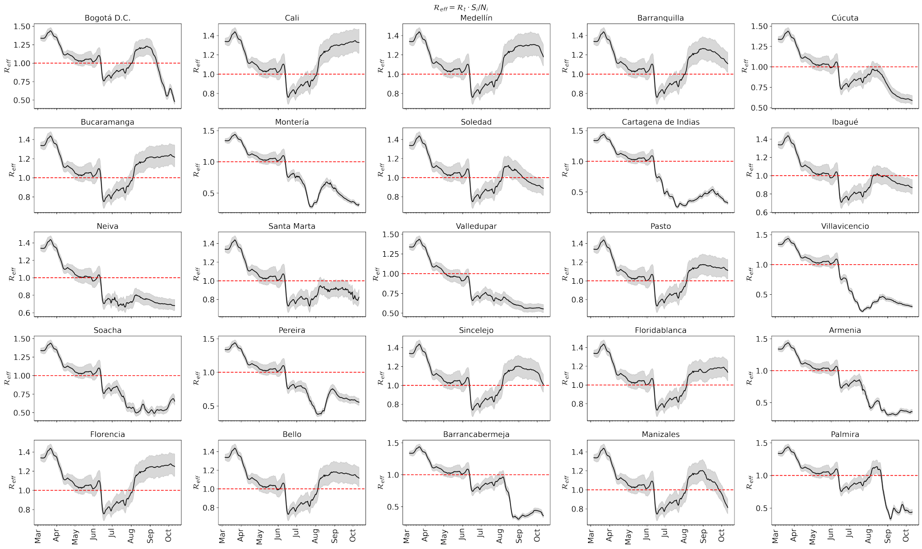

Figure S3: Each subplot show the effective reproductive number R_{eff} computed as the time varying reproduction number R_t times the estimated susceptibility (fraction of susceptible individuals in each municipality i (S_i/N_i). Black line shows median posterior estimated and gray ribbons show 95% CI. Red line show $R_{eff} = 1$ where incident cases are expected to maintain constant at the specified time.

S.7 Medellin vs Colombia report rate

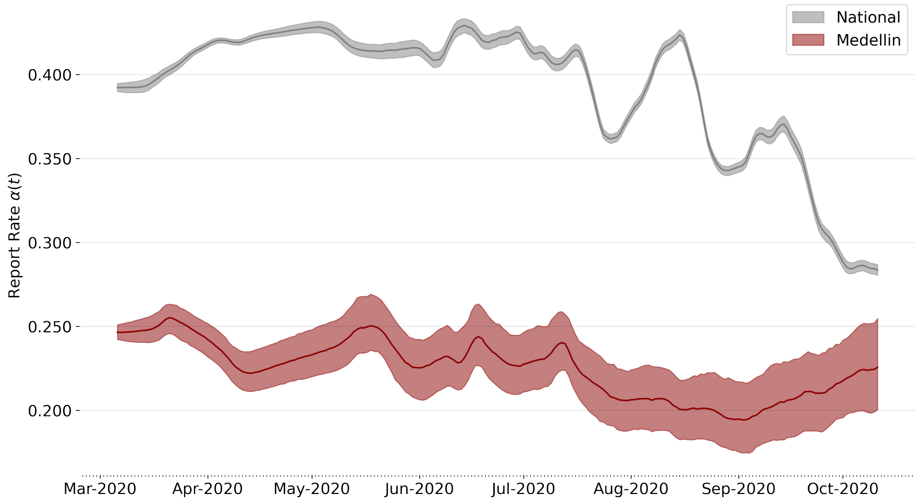

Figure S4: Report rates for Medellin and Colombia. Ribbons shows 95% CI and line shows the median, each place is indicated in the legend.

S.8 Spatial Distribution of the effective reproductive number

Spatial distribution of the effective reproductive number $\mathcal{R}_{eff}$

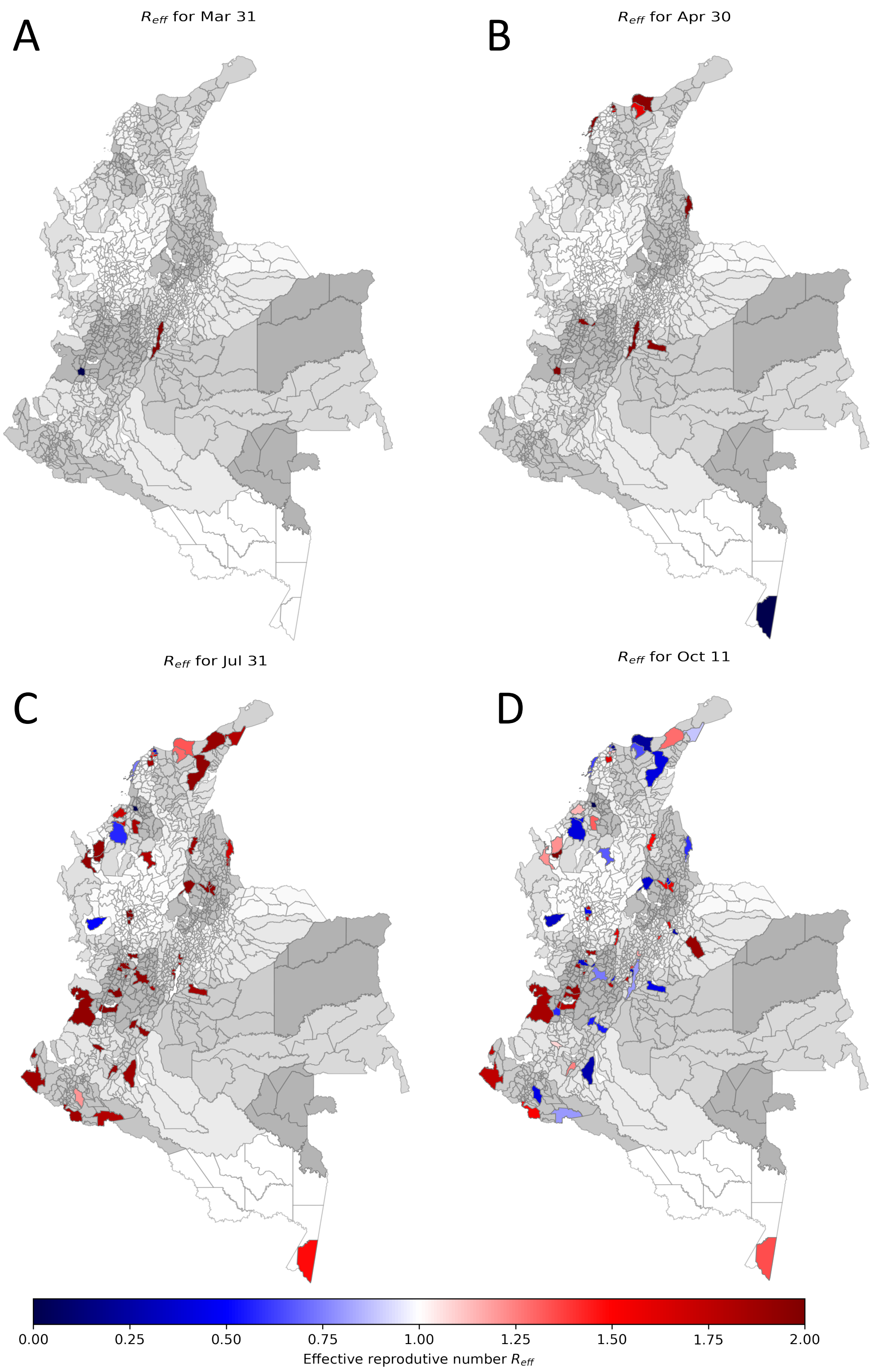

Figure S5: Effective Reproductive Number R_{eff}, computed as time-variable reproductive number times the susceptible fraction estimated for each municipality $R_{eff} = R_t \frac{S_i(t)}{N_i}$. Color key: Red colors indicate median estimates $R_{eff} > 1$ while blue colors indicate $R_{eff} < 1$ magnitude is presented in colormap. **A)** R_{eff} for municipalities with reported deaths by March 31, one month since first reported case. **B)** R_{eff} for municipalities with reported deaths by Apr 31 2020, two months since first reported case. **C)** R_{eff} for municipalities with reported deaths by July 31 2020, five months since first reported case. **D)** R_{eff} for municipalities with reported deaths by October 11 2020, 8 months since first reported case.

S.9 Sensitivity analysis

Sensitivity analysis for the parameter of the effective reproduction number. We have studied the change in the effective reproductive number to respect the parameters (the sensitivity index of $\mathcal{R}_{eff}$ of alpha for example accounts for the report rate). Rather than comparing absolute changes, we normalize the sensitivity indices in order to compare the 1% changes of parameters to see how it influences $\mathcal{R}_{eff}$.

The exact expression is:

$$E(R_t, \phi_t) = \frac{\partial R_t}{\partial \phi_i}\frac{\phi_i}{R_t}$$

Then, the analytical normalized sensitivity indexes for the parameters and it's value using the best mean posterior estimates are:

$$\begin{aligned}
E(\beta_t) &= 1\\
E(T_r) &= 1\\
E(\zeta) &= \frac{\zeta}{1-\zeta} = 0.009713[0.009593 - 0.009828] \quad 95\%CI\\
E(\alpha_t) &= \frac{\alpha_t(1-\sigma)}{(1-\alpha)\sigma + \alpha_t} = 0.442645[0.441758 - 0.443485] \quad 95\%CI\\
E(\sigma) &= \frac{\sigma(1-\alpha)}{(1-\alpha)\sigma + \alpha_t} = 0.218746[0.217655 - 0.219720] \quad 95\%CI
\end{aligned}$$

S.10 Number of infected Municipalities

Median estimates of the effective reproductive number R_{eff} for the top 5 municipalities with more reported cumulative death by October.

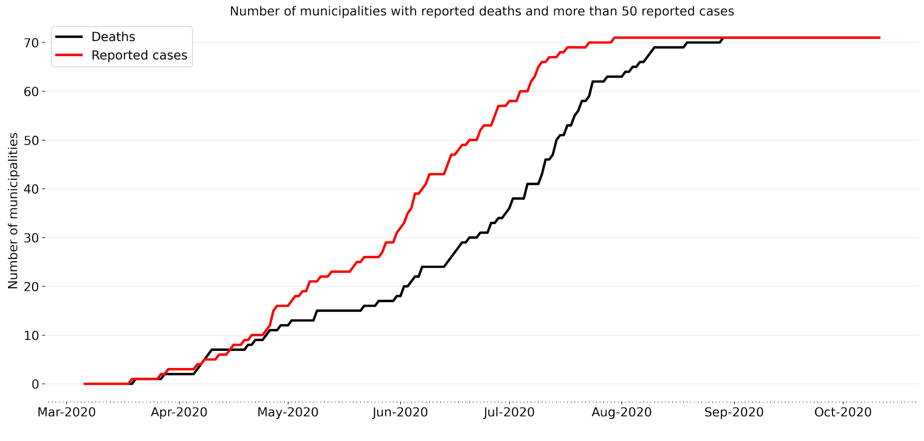

Figure S6: Number of municipalities with more than 50 reported cases (red line) and more than 1 reported death (black line).

References

[1] Pauli Virtanen, Ralf Gommers, Travis E. Oliphant, Matt Haberland, Tyler Reddy, David Cournapeau, Evgeni Burovski, Pearu Peterson, Warren Weckesser, Jonathan Bright, Stéfan J. van der Walt, Matthew Brett, Joshua Wilson, K. Jarrod Millman, Nikolay Mayorov, Andrew R. J. Nelson, Eric Jones, Robert Kern, Eric Larson, C J Carey, İlhan Polat, Yu Feng, Eric W. Moore, Jake VanderPlas, Denis Laxalde, Josef Perktold, Robert Cimrman, Ian Henriksen, E. A. Quintero, Charles R. Harris, Anne M. Archibald, Antônio H. Ribeiro, Fabian Pedregosa, Paul van Mulbregt, and SciPy 1.0 Contributors. SciPy 1.0: Fundamental Algorithms for Scientific Computing in Python. *Nature Methods*, 17:261–272, 2020.

[2] Sam Abbott, Joel Hellewell, Robin N. Thompson, Katharine Sherratt, Hamish P. Gibbs, Nikos I. Bosse, James D. Munday, Sophie Meakin, Emma L. Doughty, June Young Chun, Yung-Wai Desmond Chan, Flavio Finger, Paul Campbell, Akira Endo, Carl A. B. Pearson, Amy Gimma, Tim Russell, Stefan Flasche, Adam J. Kucharski, Rosalind M. Eggo, and Sebastian Funk. Estimating the time-varying reproduction number of SARS-CoV-2 using national and subnational case counts. *Wellcome Open Research*, 5:112, 2020.

[3] Katelyn M Gostic, Lauren McGough, Edward Baskerville, Sam Abbott, Keya Joshi, Christine Tedijanto, Rebecca Kahn, Rene Niehus, James A Hay, Pablo M. De Salazar, Joel Hellewell, Sophie Meakin, James Munday, Nikos Bosse, Katharine Sherratt, Robin M Thompson, Laura F White, Jana Huisman, Jérémie Scire, Sebastian Bonhoeffer, Tanja Stadler, Jacco Wallinga, Sebastian Funk, Marc Lipsitch, and Sarah Cobey. Practical considerations for measuring the effective reproductive number, rt. *medRxiv*, 2020.

[4] Wan Yang, Sasikiran Kandula, Mary Huynh, Sharon K. Greene, Gretchen Van Wye, Wenhui Li, Hiu Tai Chan, Emily McGibbon, Alice Yeung, Don Olson, Anne Fine, and Jeffrey Shaman. Estimating the infection-fatality risk of SARS-CoV-2 in New York City during the spring 2020 pandemic wave: a model-based analysis. *The Lancet Infectious Diseases*, 3099(20):1–10, 2020.

[5] Megan O'Driscoll, Gabriel Ribeiro Dos Santos, Lin Wang, Derek A.T. Cummings, Andrew S. Azman, Juliette Paireau, Arnaud Fontanet, Simon Cauchemez, and Henrik Salje. Age-specific mortality and immunity patterns of SARS-CoV-2. *Nature*, August 2020.

[6] Sen Pei, Marta Galanti, Teresa Yamana, and Jeffrey Shaman. Reconciling Diverse Estimates of COVID-19 Infection Rates Authors : Sen Pei , Marta Galanti , Teresa Yamana , Jeffrey Shaman Department of Environmental Health Sciences , Mailman School of Public Health , Columbia University. *Department of Environmental Health Sciences, Mailman School of Public Health, Columbia University*, pages 1–4, 2020.

[7] Ruiyun Li, Sen Pei, Bin Chen, Yimeng Song, Tao Zhang, Wan Yang, and Jeffrey Shaman. Substantial undocumented infection facilitates the rapid dissemination of novel coronavirus (SARS-CoV2). *Science (New York, N.Y.)*, 493(May):489–493, 2020.

[8] Graham C Gibson, Nicholas G Reich, and Daniel Sheldon. Real-time mechanistic bayesian forecasts of covid-19 mortality. *medRxiv*, pages 1–33, 2020.
